# High-Throughput Omics and Statistical Learning Integration for the Discovery and Validation of Novel Diagnostic Signatures in Colorectal Cancer

**DOI:** 10.3390/ijms20020296

**Published:** 2019-01-12

**Authors:** Nguyen Phuoc Long, Seongoh Park, Nguyen Hoang Anh, Tran Diem Nghi, Sang Jun Yoon, Jeong Hill Park, Johan Lim, Sung Won Kwon

**Affiliations:** 1 College of Pharmacy and Research Institute of Pharmaceutical Sciences, Seoul National University, Seoul 08826, Korea; phuoclong@snu.ac.kr (N.P.L.); 2018-23140@snu.ac.kr (N.H.A.); supercanboy@snu.ac.kr (S.J.Y.); hillpark@snu.ac.kr (J.H.P.); 2 Department of Statistics, Seoul National University, Seoul 08826, Korea; inmybrain@snu.ac.kr (S.P.); johanlim@snu.ac.kr (J.L.); 3 School of Medicine, Vietnam National University, Ho Chi Minh 70000, Vietnam; trandiemnghi@gmail.com

**Keywords:** colorectal cancer, transcriptomics, diagnosis, biomarker, machine learning, variable selection

## Abstract

The advancement of bioinformatics and machine learning has facilitated the discovery and validation of omics-based biomarkers. This study employed a novel approach combining multi-platform transcriptomics and cutting-edge algorithms to introduce novel signatures for accurate diagnosis of colorectal cancer (CRC). Different random forests (RF)-based feature selection methods including the area under the curve (AUC)-RF, Boruta, and Vita were used and the diagnostic performance of the proposed biosignatures was benchmarked using RF, logistic regression, naïve Bayes, and k-nearest neighbors models. All models showed satisfactory performance in which RF appeared to be the best. For instance, regarding the RF model, the following were observed: mean accuracy 0.998 (standard deviation (SD) < 0.003), mean specificity 0.999 (SD < 0.003), and mean sensitivity 0.998 (SD < 0.004). Moreover, proposed biomarker signatures were highly associated with multifaceted hallmarks in cancer. Some biomarkers were found to be enriched in epithelial cell signaling in *Helicobacter pylori* infection and inflammatory processes. The overexpression of *TGFBI* and *S100A2* was associated with poor disease-free survival while the down-regulation of *NR5A2*, *SLC4A4*, and *CD177* was linked to worse overall survival of the patients. In conclusion, novel transcriptome signatures to improve the diagnostic accuracy in CRC are introduced for further validations in various clinical settings.

## 1. Introduction

Colorectal cancer (CRC) is the fourth most frequently diagnosed cancer [1]. The 5-year survival rate in case of early detection is 90%, but only 39% of CRC cases are diagnosed at a localized stage [2]. The majority of CRCs slowly proceed from adenomas through curable localized malignant lesions to metastatic CRC over a lengthy period, opening up a large window for screening and early detection of this disease [3,4]. The diagnosis of CRC covers system-level investigation using imaging and biomarkers with the help of emerging innovative technologies and computational diagnostic models [5]. Invasive colonoscopy and sigmoidoscopy are current first-line screening tests for detection of adenomas and CRCs in the total and distal colon, respectively [3]. Computed tomography colonography (CTC), despite being noninvasive, is limited to morphologic imaging and may misdiagnose remnant stool as polyps and provide a false-positive result [6]. CTC also experiences a lower sensitivity for nonpolypoid than polypoid neoplasia [7]. In this context, blood-borne biomarkers appear to be an alternative with minimal invasiveness. Over years, carcinoembryonic antigen (CEA), carbohydrate antigen (CA) 19-9, CA 242, or their combination have been proved to be related to the diagnosis and prognosis of CRC [8]. However, the level of CEA also increases in other malignancies such as ovarian cancer, pancreatic cancer, and even in benign conditions like inflammatory bowel disease [9]. Along with the advanced knowledge in high-throughput techniques and bioinformatics tools, novel and robust omics-based biomarkers have been developed and integrated with traditional biomarkers with the hope of improving the diagnosis and prognosis of cancer and may be applicable to CRC [10,11].

Several systematic approaches and guidelines have been proposed for selecting important biomarker candidates across experiments [12]. In the circumstance that a single gene expression analysis might not provide a reliable and generalizable conclusion, the quantitative analysis of the combined datasets from multiple sources and technologies appears to be an efficient solution to increase the sample size and enhance the statistical power and thus possibly helps identify more clinically relevant biomarkers [13,14]. Choosing sufficient control samples and appropriate data mining techniques, removing batch effects across different platforms and studies are required to ensure a robust process of biomarker discovery and validation [15]. Furthermore, state-of-the-art feature selection methods and supervised classification techniques, if utilized properly, will be capable of producing highly reliable predictive gene signatures for the accurate diagnosis of the diseases of interest [16]. Among available feature selection approaches, random forest (RF) and derivative methods have been proved a suitable variable selection platform for heterogeneous and high-dimensional data [17]. However, between the Gini variable importance measures (VIM) and the permutation VIM, the permutation VIM method is unbiased and does not prefer certain predictor over others compared with the Gini VIM [18]. Janitza et al. devised a new method, the area under the curve (AUC) of the random forests or AUCRF, to assign importance scores to variables based on the area under the curve to address a classification problem with class imbalance [18]. They first recognized that the standard permutation importance scores depended on difference in error rates caused by permutation of a variable and suggested to use an alternative performance measure, the AUC, instead of an error rate [19]. Boruta trained the RF classifier on the extended dataset containing the initial dataset and its copy. The main idea of Boruta is to compare the importance of the real features with that of the duplicated shadow features [20]. It regards a variable unimportant that has a significantly less importance score than all of the simulated variables, which will be taken out of the list of attributes in a subsequent iteration. Their algorithm goes in a sequential way to mark importance of all variables at the end. Lastly, compared to the standard algorithm from Breiman [19] where out-of-bag samples are tested to compute scores, the recently published Vita algorithm [21], inspired by the cross-validation, splits samples into k sets (e.g., k = 2 for the sake of brevity) to evaluate importance scores; one for growing a forest and the other for computing permutation variable importance. It was developed in the R package under the name of *vita*. A strong point of this method is that it has comparable power with better stability in feature selection while requiring a significantly shorter computation time, which is appropriate to handle large datasets [21].

In the current study, we implemented AUCRF, Boruta, and Vita for selecting novel biomarker candidates from microarray-based gene expression of 340 CRC patients and 134 paired noncancerous controls. Afterward, only genes that are early dysregulated in the tumorigenesis of CRC, at the adenoma stage, were selected to produce the final sets of biomarker candidates. Various prediction models of the three biosignatures were then built, validated, and compared using 762 CRC and non-cancerous samples from processed and batch effects removed whole-transcriptome shotgun sequencing (RNA-seq)-based gene expression derived from The Cancer Genome Atlas (TCGA) and Genotype-Tissue Expression (GTEx) cohorts. Finally, the mode of action in cancer hallmarks and impact of the biomarker candidates in the prognosis of the patients were also examined. Our comprehensive analysis using statistical learning on gene expression patterns successfully introduced and validated novel biomarkers. Proposed predictive transcriptome signatures revealed strong classification power and might be utilized to support the diagnostic decision making in clinical practice to improve the management of CRC patients. Our study covered the biomarker discovery, prediction model building and validation, and established a foundation for the clinical validation step.

## 2. Results

### 2.1. The Construction of Genetic Biosignatures for the Diagnosis of Colorectal Cancer

**Figure 1:**
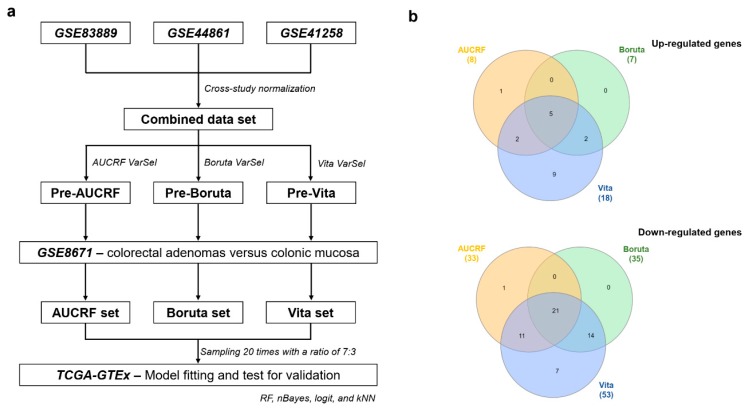
Workflow of the biomarker candidate selection. (**a**) The process of selecting and validating diagnostic candidates with three different variable selection algorithms. (**b**) The Venn diagram demonstrating the relationships of selected biomarker candidates among three methods. VarSel: Variable selection; RF: Random Forest; AUCRF: the area under the curve (AUC)-RF; nBayes: naïve Bayes; logit: logistic regression; kNN: k-nearest neighbors.

Samples from heterogeneous ethnics with different patients’ bio-parameters were utilized in data-driven biomarker discovery. The data set GSE44861 comprised 56 CRC tissues and 55 adjacent noncancerous tissues from the United States [22]. The data set GSE41258 had 183 CRC and 44 adjacent noncancerous tissues from the United States between 1992 and 2004 [23]. The data set GSE83889 contained 101 CRC tissues and 35 non-neoplastic mucosal tissues from all patients with stage III of CRC from Korea [24]. They were all batch effects corrected and combined into a single data set for the variable selection process (Figure 1a). In addition, a data set containing 32 paired adenomas and colonic mucosa from an Italy cohort (GSE8671) was applied as an attempt to ensure that our biomarker candidates were dysregulated in early stages of the colorectal tumorigenesis [25]. More information on each data set can be found in Table 1 and the original publications.

According to the results, 747, 179, and 953 genes passed the selection criteria for AUCRF, Boruta, and Vita, respectively. They were redundant and not representative for the early stage of the disease, which is essential for diagnostic biomarkers. Hence, we filtered them with dysregulated genes in adenomas. After being filtered, only 41 (8 up-regulated, 33 down-regulated), 42 (7 up-regulated, 35 down-regulated), and 71 (18 up-regulated, 53 down-regulated) genes remained. Interestingly, the Boruta-derived biosignature was a part of the Vita-derived biosignature. There were 5 up-regulated genes and 21 down-regulated genes in common among three methods. Thus, AUCRF possessed only one distinct up-regulated gene and one distinct down-regulated gene (Figure 1b). *ADH1C*, *GBA3*, and *CD177* were not found in the RNA-seq and were eventually removed from the classification and validation process. Ultimately, there were 39, 41, and 68 individual markers from AUCRF, Boruta, and Vita, respectively.

### 2.2. Classification Model Development, Validation, and Comparisons of the Three Genetic Biosignatures

#### 2.2.1. Data Exploration and Visualization

**Figure 2:**
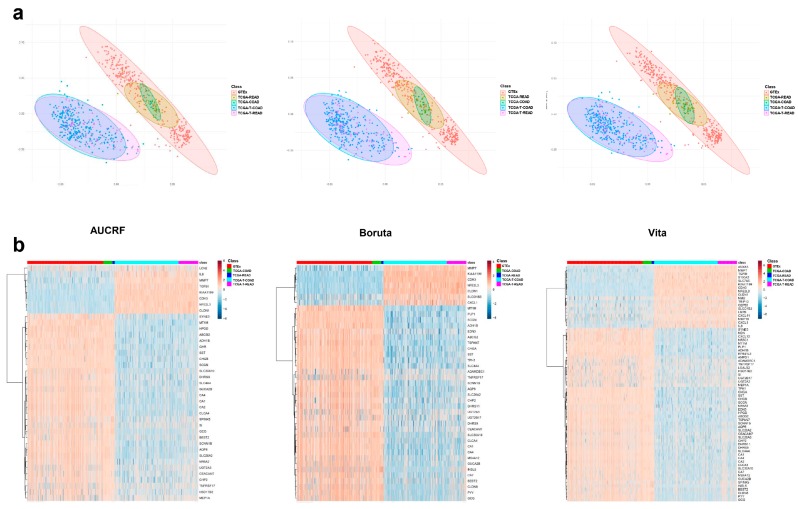
Data exploration of three sets of biomarker candidates. (**a**) Principal component analysis of three sets of biomarker candidates between cancer samples and non-cancerous samples. (**b**) Heatmap analysis of three sets of biomarker candidates between cancer samples and non-cancerous samples. TCGA-READ: normal rectum, TCGA-COAD: normal colon, TCGA-T-READ: rectum adenocarcinoma, TCGA-T-COAD: colon adenocarcinoma, GTEx: normal colon and rectum.

RNA-seq data set including CRC samples (TCGA-T-coad and TCGA-T-read) noncancerous samples (GTEx, TCGA-coad, and TCGA-read) was subjected to data exploration and visualization. It is worthy to mention that no or limited batch effects were shown in the original processed RNA-seq data set (Figure S1). After principal component analysis (PCA), the two first principal components were used to visualize all samples in a 2D plot. As shown in Figure 2a, AUCRF-derived signature, Boruta-derived signature, and Vita-derived signature, all showed excellent separations. Some outliers were observed, whichsuggested those cases might be misclassified in the classification using supervised learning models. In heatmap analysis, we could observe a clear contrast between CRC and non-cancerous controls. Noticeably, down-regulated genes were much common than the up-regulated genes in all signatures. Among down-regulated genes, there were two sub-patterns; one seems to be more down-regulated than another (Figure 2b). Finally, the gene expression patterns of colon and rectum in either cancer or non-cancerous samples were highly comparable.

**Figure 3:**
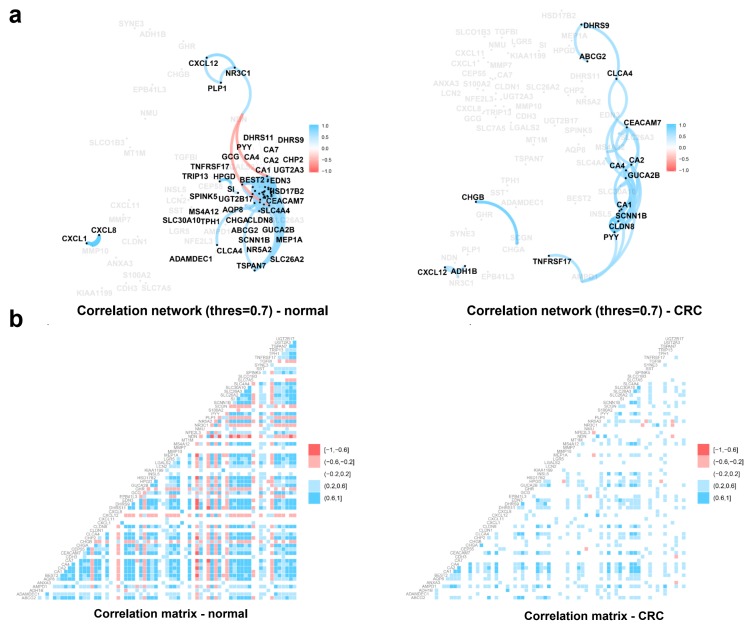
Correlation analysis of biomarker candidates of cancer samples and non-cancerous samples. (**a**) Correlation network of biomarkers in cancer samples and non-cancerous samples. Blurred edges in the network were the ones with correlation strength (in absolute value) below the cut-off value 0.7. The blue color indicates positive correlations while red color indicates negative correlations (**b**) Correlation matrix of biomarkers in cancer samples and non-cancerous samples.

Potential biomarker candidates derived from conventional statistical biomarker selection are often highly collineared. Thus, we conducted correlation network analysis to check whether the multicollinearity occurred among predictors in three signatures: AUCRF-derived signature, Boruta-derived signature, and Vita-derived signature. As shown in the correlation network, the predictors are highly correlated to each other in the non-cancerous group but possess a small correlation network in CRC group (Figure 3a). The correlation scores from one to another of the predictors are presented in the correlation matrix plot (Figure 3b).

#### 2.2.2. Model Fitting and Validation

We performed computational experiments with the aid of machine learning techniques built upon three sets of biosignatures. Due to a paucity of external datasets, we trained classification models and validated them by dividing available data randomly, which was repeated 20 times. Tuning parameters, if relevant, of statistical models were tuned through 10-fold cross-validation with 5 times repetitions.

**Figure 4:**
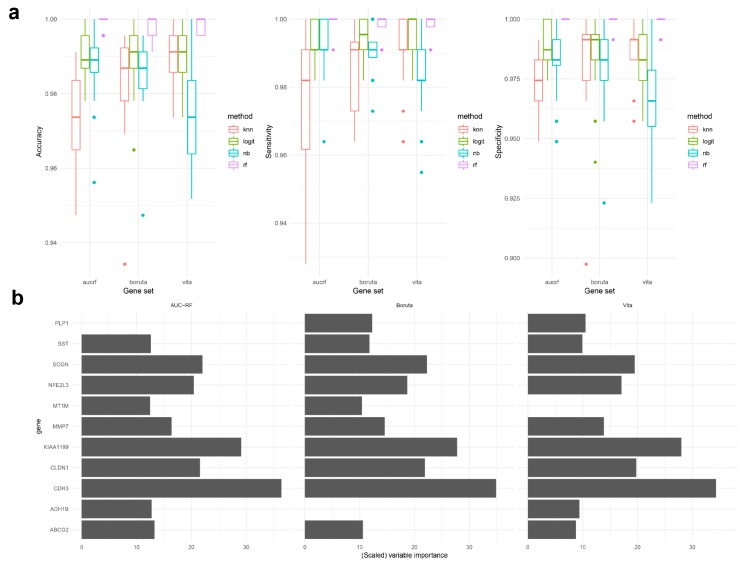
Performance metrics of classification models and variable importance scores from three tested signatures. (**a**) Accuracy, sensitivity, and specificity of various machine learning classification models. (**b**) Top 10 most important candidates of the random forests models.

RF, our primary classifier, showed highly promising performance at the validation step regardless of gene sets (Figure 4a); mean accuracy 0.998 (Standard deviation (SD) < 0.003), mean specificity 0.999 (SD < 0.003), and mean sensitivity 0.998 (SD < 0.004). In other words, only a small subset of biomarkers could distinguish every sample almost perfectly except one or two of a thousand. In particular, it can be seen from Figure 4b that eight of each gene sets commonly functioned with higher importance to the classification task than the others, which implies greater attention should be paid to them in consequent experiments. Other measures such as F1 score and Cohen’s kappa coefficient were used in evaluation, see Figure S2. Logistic regression, naïve Bayes, and kNN were also analyzed in the same fashion as benchmark methods. Hence, total 12 models (four classifiers in three gene sets) were tested to produce 20 sets of performance measures from 20 test datasets, which are summarized in Figure 4a. In a nutshell, their summary statistics were shown as follows; mean accuracy 0.973–0.990 (SD < 0.015), mean specificity 0.967–0.988 (SD < 0.017), mean sensitivity 0.975–0.995 (SD < 0.021). These comparative results to those from RF indicated that gene selection would be more essential than choice of classifiers. Moreover, the fact that such a simple regression model, or logistic regression, even worked reasonably well without tuning agrees with the preceding argument.

Sensitivity analyses were conducted to inspect the robustness of our findings. First, we examined the effect of balancing the proportion of samples in each group. Second, we tested if the unbalance between cancer and non-cancerous samples had a significant impact on the performance of the predictors by training and testing samples derived only from TCGA cohorts. Finally, we tested if the performance was heavily dependent on the dominance of non-cancerous samples from GTEx. Interestingly, there was not much difference regarding the prediction powers of utilized models in these three designs from that of the main analyses. Moreover, we found that the biomarker set derived from the Vita method gave more robust results than those from AUC-RF or Boruta (Figures S3 and S4).

### 2.3. Functional Analysis of Individual Biomarkers in Colorectal Cancer

We conducted pathway enrichment analysis using 73 individual biomarkers (19 up-regulated and 54 down-regulated) that appeared in at least one biosignature to get better insights into the associated biological processes. There were only eight enriched pathways in the up-regulated group. *CXCL1* and *CXCL8* that are associated with colorectal cancer risk and overall survival, appeared to be included in epithelial cell signaling in *Helicobacter pylori* (*H. pylori*) infection, chemokine signaling pathway and cytokine-cytokine receptor interaction [27]. There were, however, 55 enriched pathways in the down-regulated group and they were from various biological processes. More details can be found in Table S1. In addition, we conducted a text mining experiment dedicated to elucidate the associated hallmarks of cancer of 73 individual biomarkers. In the overexpressed group, 14 of them were associated with at least five hallmarks. On the other hand, 14 of the silencing biomarkers were associated with at least 5 hallmarks. Especially, among this list, the numbers of up-regulated and down-regulated genes which were reported in CRC studies are 14 and 28, respectively. Finally, available evidence of hallmarks immune destruction, cellular energetics, and replicative immortality was somehow limited. More information is shown in Table S2.

### 2.4. Prognostic Assessment of Individual Biomarkers in Colorectal Cancer

**Figure 5:**
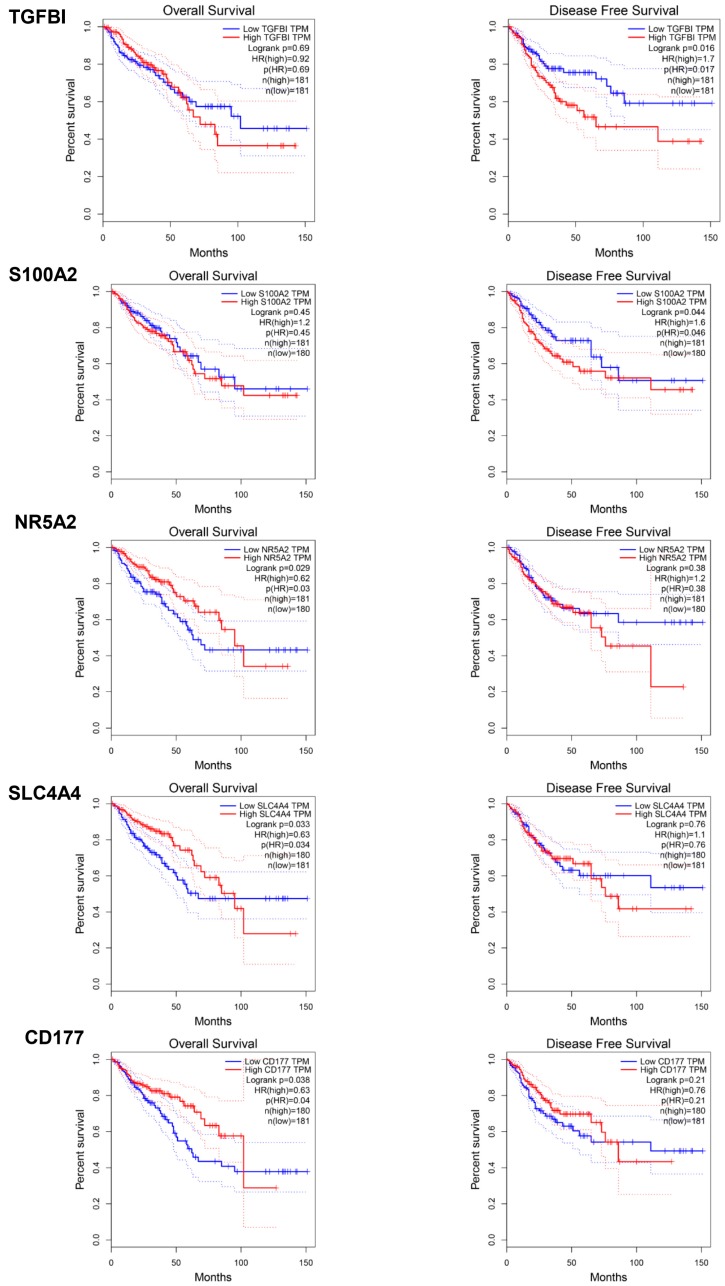
Overall survival and disease-free survival analysis of *TGFBI*, *S100A2*, *NR5A2*, *SLC4A4*, and *CD177*.

The combined cohorts of CRC from TCGA Colon Adenocarcinoma (TCGA-T-COAD) and TCGA Rectum Adenocarcinoma (TCGA-T-READ) were used for the survival analysis. The relationships between gene expression and both overall survival (OS) and disease-free survival (RFS) were examined (Table S3). Among total 19 individual up-regulated biomarkers belonging to at least one signature, *ANXA3* (OS), *IL8* (*CXCL8*) (OS), and *CXCL11* (DFS) were associated with the preferred outcome of CRC patients. On the contrary, the up-regulation of *TGFBI* (DFS) and *S100A2* (DFS) was related to the poor outcome of the patients. When investigating 54 individual down-regulated biomarker candidates, the deletion of *ADAMDEC1*, *CEACAM7*, *GCG*, *AQP8*, *BEST2*, *SLC26A2*, and *SLC26A3* was all related to a better OS of the patients. Finally, down-regulations of *NR5A2*, *SLC4A4*, and *CD177* were the indicators of poor OS prognosis of the CRC patients. The Kaplan-Meier plots of *TGFBI*, *S100A2*, *NR5A2*, *SLC4A4*, and *CD177* are shown in Figure 5. The above results suggested that our diagnostic signatures had a confined impact on predicting the prognosis of the CRC patients.

## 3. Discussion

The explosion of high-throughput data and literature database has facilitated integrative data- and knowledge-driven biomarker exploration and validation processes [28]. Pioneering studies using large-scale gene expression to discover novel biomarkers mainly applied univariate statistical methods with proper corrections. This approach is relatively straightforward and suitable for single data set. However, along with the accumulation of the high-throughput data and the rapid advancement of statistical learning algorithms, machine learning-based approaches are gaining popularity due to their outstanding reproducibility and robustness [29]. The use of microarray-based transcriptome data sets suffers from various technological and practical limitations as well as small size of samples that may result in irreproducible conclusions [30]. In addition, lack of cross-study validation using similar or different platforms also diminishes the translation from the discovery phase to the real-world applications [31]. Of note, a desirable set of biomarkers should hold its consistency when being tested against various technical and experimental factors. Collectively, a solid workflow and statistical approach for introducing diagnostic, prognostic, and predictive biomarkers are in urgent need.

The predictive transcriptome signatures derived from an early phase translational research may be further developed and eventually implemented to assist the clinicians in making clinical decision. For instance, a previous investigation aimed to establish a novel profile from 12 public microarray data sets using 17 known CRC-associated genes suggested a seven-gene model with encouraging results for further investigations with blood-based assays [32]. Another study on 31 CRC and 33 non-tumoral samples also suggested the possibility of a small biomarker panel [33]. Our study, on the other hand, implemented a novel data-driven approach that allows us to overcome the fundamental limitations of an investigation of high-throughput gene expression data. First, we collected available microarray data from biologically comparable CRC and adjacent noncancerous tissues and later combined them into a larger data set for variable selection exploiting state-of-the-art feature selection techniques. Moreover, the use of paired samples helps the selection process to target mostly on the biological differences and reduces various confounding factors in affecting the introduction of novel biomarker candidates. Second, we applied a so-called ‘filter’ to initial sets of biomarker candidates of which we were able to selectively focus on the early dysregulated genes during the tumorigenesis of CRC. Subsequently, the mature biosignatures may be more reliable for the purpose of early detection of CRC in clinical settings. Third, we externally tested and validated novel biosignatures using relatively large-size samples from RNA-seq data set with various supervised learning classification techniques. It is of importance to note that the three sets of biomarkers which resulted from three cutting-edge variable selection methods possessed highly comparable performance when combined with all tested classification models. Finally, the biologically relevant individual biomarker candidates were revealed using data mining techniques and pathway enrichment analysis. Overall, we successfully introduced, validated, and compared the performance of proposed diagnostic biomarkers. Noticeably, *SST*, *SCGN*, *NFE2L3*, *MMP7*, *KIAA1199 (CEMIP)*, *CLDN1*, *CDH3*, and *ADH1B* were consistently among the most important features from the RF models of AUCRF-derived signature, Boruta-derived signature, and Vita-derived signature. Importantly, the biomarker list derived from Vita exhibited an extremely stable performance in all tested analyses. In addition, the biological functions of the biomarkers in cancer were also revealed to suggest further mechanistic and translational studies to move forward to the applications. Network-based pathway enrichment analysis suggested that the proposed predictors and closely associated genes are enriched in several well-known cancerous processes, such as chemokine signaling pathway or cytokine-cytokine receptor interaction. Moreover, some of these genes belong to the epithelial cell signaling in *H. pylori* infection. These pathways, together with previous clinical and laboratory findings, indicate a potential oncogenic interaction between *H. pylori* and colorectal mucosa [34,35,36]. Thus, we suggest that the analysis of large-scale omics data may provide new insights into the subject of ongoing research that whether *H. pylori* infection causally triggers colon tumor formation.

Notwithstanding, our study has several limitations. For instance, there is a lack of the detailed patients’ bio-parameters, which may affect the measurement of the potential biomarkers [16]. However, the study design allowed us to use the paired samples for selecting potential biomarker candidates and a diverse group of CRC and noncancerous samples for testing their performance. The paired sampling method reduces individual-specific and anatomical site-specific effects [37]. Also, the implementation of machine learning-based approach reduces the possibility of explaining how the models make their decisions, which leads to a black-box scenario. This may be partly explained using emerging explaining techniques, such as local interpretable model-agnostic explanations—a local interpretability of complex response functions, when a practical model is clinically used [38]. Finally, a translational study using biofluids and/or feces with our proposed signatures is necessary to facilitate the development of less invasive diagnostic techniques.

We successfully introduce a solid workflow for biomarker discovery and validation that is able to overcome some current technical and experimental limitations of high-throughput gene expression platforms. Novel transcriptome signatures that may improve the diagnostic accuracy in colorectal cancer are introduced and subjected to additional translational evaluation and application. Further studies are required to address the limitations of our study and validate proposed predictive signature in various clinical settings.

## 4. Materials and Methods

### 4.1. Patients and Samples

Colorectal cancer and matched non-neoplastic mucosal tissues of GSE83889 (HumanTH-12 V4.0), GSE44861 (HG-U133A), and GSE41258 (HG-U133A) were collected from Gene Expression Omnibus (GEO) and served as the samples for selecting novel biomarker candidates. In addition, colorectal adenomas and corresponding colonic mucosa samples from GSE8671 (HG-U133 Plus 2) were collected to analyze the dysregulated genes in colorectal adenomas. To measure the classification performance of the biomarker candidates, the batch effects corrected RNA-seq data of TCGA (TCGA-READ and TCGA-COAD) and GTEx that are available from literature were utilized [26]. The use of authentic public data and/or commercial samples without sensitive information of the patients for this study was waived through a full ethical application by the Institutional Review Board of Seoul National University (SNU 18-01-004).

### 4.2. Data Pre-Processing

Affymetrix and Illumina microarray derived data sets were pre-processed by either affy package or lumi package when applicable. For batch effects removal, we applied the Empirical Bayes cross-study normalization method implemented in NetworkAnalyst [39].

### 4.3. Gene Expression Analysis

Differential gene expression analysis with a blocking factor as the second metadata set was applied to analyze gene expression of the paired design samples using NetworkAnalyst. The mean was used as the gene-level summarization in the annotation step. Data filtering was performed to remove genes with 15% lowest variance. A Log_2_ fold change of 1 was applied as the feature selection in adenoma versus normal tissue data set. The analysis was conducted using NetworkAnalyst [39].

### 4.4. Data Exploration and Visualization

Principal component analysis (PCA) was used for gene expression (log transformed) data visualization and to detect potential outliers prior to the class assignment analysis using ggfortify 0.4.5 [40]. A heatmap was also applied to highlight the difference in gene expression (log transformed and scaled) level between two comparison groups using MetaboAnalyst 4.0 [41]. For features, the distance measure was Euclidean and the clustering algorithm was Ward. A Venn diagram was illustrated by InteractiVenn [42].

### 4.5. Variable Selection Method

Variable selection was conducted using the combined microarray data set of CRC and adjacent noncancerous samples. AUCRF was implemented using party package 1.3 with a selection criterion of genes with the top 20% highest importance score. Boruta was implemented using the Boruta package version 5.3.0 with a selection criterion of ‘*p*-value’ of 0.01. Vita was implemented using the Vita package 1.0.0 with a selection criterion of ‘threshold for *p*-values’ of 0 [43].

### 4.6. Classification Model Fitting and Validation

A random forest was chosen as our main decision maker in the task of classification between cancer patients and non-cancerous controls since all variable selection methods were based on it. The model was constructed and validated using 762 transcriptome samples by splitting training and test samples by the ratio of 7:3 while the relative proportion of non-cancer and cancer groups was kept balanced within each dataset. With a training data set, the number of variables used in each split of a tree was tuned by random grid search through 5 times repeated 10-fold cross-validation, and the consequent final model determined by AUC was evaluated on the test set to gauge various performance measures. We repeated this procedure 20 times to reduce uncertainty in random splitting. To check how robust three signatures are in prediction, three commonly used classification techniques (logistic regression, naïve Bayes, and kNN models) were applied in the same manner described above. Logistic regression does not have parameters to be tuned, but naïve Bayes has the use of kernel to be used, and kNN should be tuned by the number of neighbors.

A unified framework of model training and validation was applied using the Classification and Regression Training (caret) package 6.0.80 [44]. Accuracy, sensitivity, specificity, kappa, and F1 score on the test sets of all models were reported and visualized using ggplot2 package [45].

### 4.7. Correlation Network Analysis

Correlation analysis was conducted for gene expression level based on the R package *corrr* [46], which projects the distances matrix obtained from the correlation matrix into a low-dimensional space so that graphical visualization is facilitated. The core of this analysis is the multidimensional scaling, a dimension reduction technique that aims to find low-dimensional coordinates in Euclidean space while minimizing distortion of information in distances. We blurred edges in the network with correlation strength (in absolute value) below the cut-off value 0.7 and nodes as well without remaining edges.

### 4.8. Survival Analysis

The overall survival and disease-free survival were investigated using the Kaplan-Meier method with a log-rank test. The median value was utilized to set the high and low gene expression level groups. The hazard ratio and the 95% confidence interval information were measured. The process was implemented in the GEPIA web tool [47].

### 4.9. Functional Analysis

Cancer Hallmarks Analytics Tool (CHAT) was applied to find the association between biomarker candidates and documented evidence of these molecules in hallmarks of cancer [48]. Network-based pathway enrichment analysis was conducted using OmicsNet [41], and the database of Kyoto Encyclopedia of Genes and Genomes (KEGG) was applied for the pathway annotation.

### 4.10. Statistical Significance Level

A *p*-value of 0.05 was used as the cut-off for significance. A false discovery rate (Benjamini-Hochberg method) threshold of 0.10 was utilized in pathway enrichment analysis and of 0.05 for all other multiple hypothesis tests. Finally, R statistic 3.5.1 was used to implement the statistical analysis except otherwise stated [34].

## Figures and Tables

**Table 1:** Characteristics of the included data set for variable selection and model fitting.

Section	Comparison	Author	Data Set	Year	Platform	Samples		
Variable selection	*Cancer versus Normalcy*					**Normalcy**	**Adenoma**	**Cancer**
		Ryan BM et al. [22]	GSE44861 ^1^	2013	Affymetrix U133A	55		56
		Sheffer M et al. [23]	GSE41258 ^1^	2012	Affymetrix U133A	44		183
		Kwon Y et al. [24]	GSE83889 ^1^	2016	Illumina HumanHT-12 V4.0	35		101
	*Adenoma versus Normalcy*	Marra G et al. [25]	GSE8671 ^1^	2007	Affymetrix U133 2.0	32	32	
Model fitting and validation		**Author**	**Type**				**Non-malignancy**	**Cancer**
	*Cancer versus Non-malignant*	TCGA, GTEx [26]	coad-rsem-fpkm-tcga, coad-rsem-fpkm-tcga-t, read-rsem-fpkm-tcga, read-rsem-fpkm-tcga-t, colon-rsem-fpkm-gtex				390 ^2^	372 ^3^

^1^ Paired samples; ^2^ 41 from coad-rsem-fpkm-tcga, 10 from read-rsem-fpkm-tcga, 339 from GTEx; ^3^ 285 from coad-rsem-fpkm-tcga-t, 87 from read-rsem-fpkm-tcga-t.

## Supplementary Materials

Supplementary Materials can be found at http://www.mdpi.com/1422-0067/20/2/296/s1. Figure S1: Principal component analysis of original curated data of TCGA and GTEx RNA-seq from [26]. (a) Raw data. (b) Normalized data. (c) Normalized and batch effects removal data. TCGA-READ: normal rectum, TCGA-COAD: normal colon, TCGA-T-READ: rectum adenocarcinoma, TCGA-T-COAD: colon adenocarcinoma, GTEx: normal colon and rectum. Figure S2: F1 score and Cohen’s kappa coefficient of all classification models of three sets of biomarkers. Figure S3: Classification performance with respect to the balancing proportion of each data set. Figure S4: Classification performance of TCGA-derived data sets only and TCGA-derived cancer samples versus GTEx non-cancerous samples. Table S1: Significantly enriched pathways of 19 up-regulated and 53 down-regulated potential biomarkers. Table S2: The summary of Cancer Hallmarks Analytics Tool analysis for 19 up-regulated and 54 down-regulated genes. Table S3: Survival analysis of 19 up-regulated and 54 down-regulated genes.

## Abbreviations

CRC	Colorectal cancer
TCGA	The Cancer Genome Atlas
VIM	Variable importance measures
AUC	Area under the receiver operating characteristic curve
RF	Random forest
AUCRF	The area under the receiver operating characteristic curve of the random forest
GTEx	Genotype-Tissue Expression
PCA	Principal component analysis
CHAT	Cancer Hallmarks Analytics Tool
KEGG	Kyoto Encyclopedia of Genes and Genomes
GEO	Gene Expression Omnibus
