# Supplementary material for: High-Throughput Omics and Statistical Learning Integration for the Discovery and Validation of Novel Diagnostic Signatures in Colorectal Cancer
Source: Int J Mol Sci. 2019 Jan 12;20(2):296. doi: 10.3390/ijms20020296 (PMC6358915; doi:10.3390/ijms20020296)
Supplement: Supplementary file 1 [file ijms-20-00296-s001.pdf]

International Journal of *Molecular Sciences*

MDPI

Article

High-throughput omics and statistical learning integration for the discovery and validation of novel diagnostic signatures in colorectal cancer

Nguyen Phuoc Long[1], Seongoh Park[2], Nguyen Hoang Anh[1], Tran Diem Nghi[3], Sang Jun Yoon[1], Jeong Hill Park[1], Johan Lim[2], Sung Won Kwon[1,*]

[1] College of Pharmacy and Research Institute of Pharmaceutical Sciences, Seoul National University, Seoul 08826, Republic of Korea.
[2] Department of Statistics, Seoul National University, Seoul 08826, Republic of Korea.
[3] School of Medicine, Vietnam National University, Ho Chi Minh 70000, Vietnam.

* Correspondence: Sung Won Kwon (swkwon@snu.ac.kr); Tel.: +82-2-880-7844

Figure S1: Principal component analysis of original curated data of TCGA and GTEx RNA-seq.
(a) Raw data. (b) Normalized data. (c) Normalized and batch effects removal data.
TCGA-READ: normal rectum, TCGA-COAD: normal colon, TCGA-T-READ: rectum adenocarcinoma, TCGA-T-COAD: colon adenocarcinoma, GTEx: normal colon and rectum.

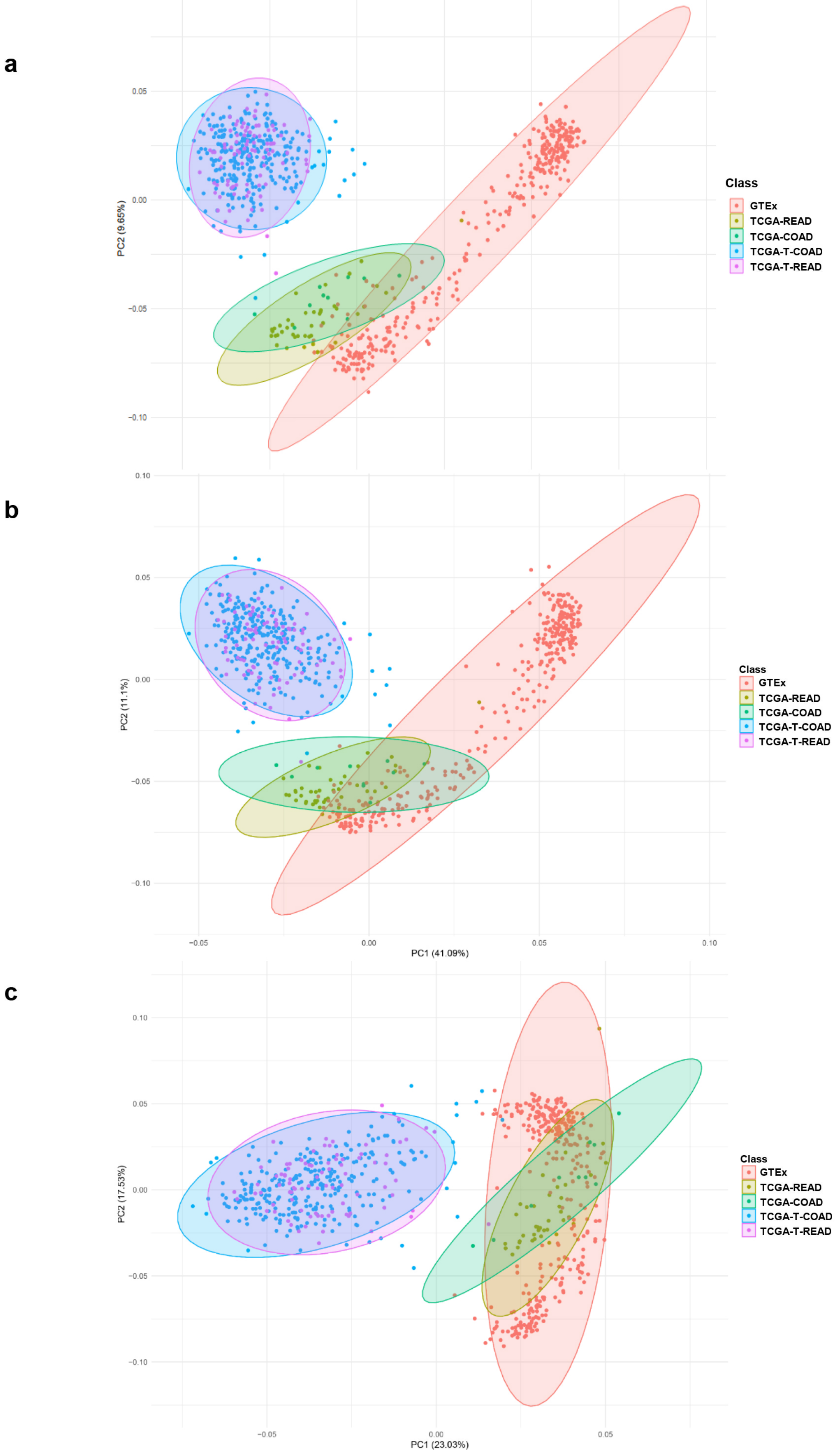

Figure S2: F1 score and Cohen's kappa coefficient of all classification models of three sets of biomarkers.

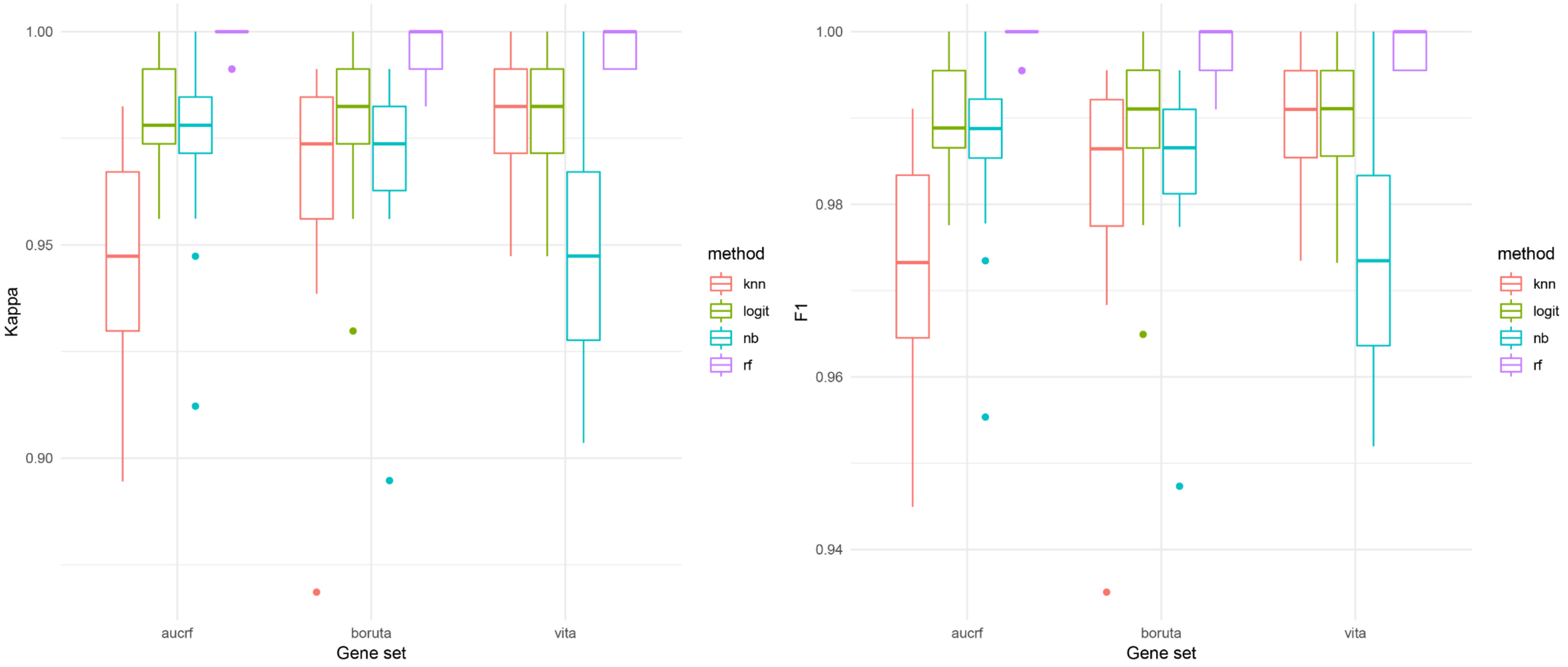

Figure S3: Classification performance with respect to the balancing proportion of each data set.

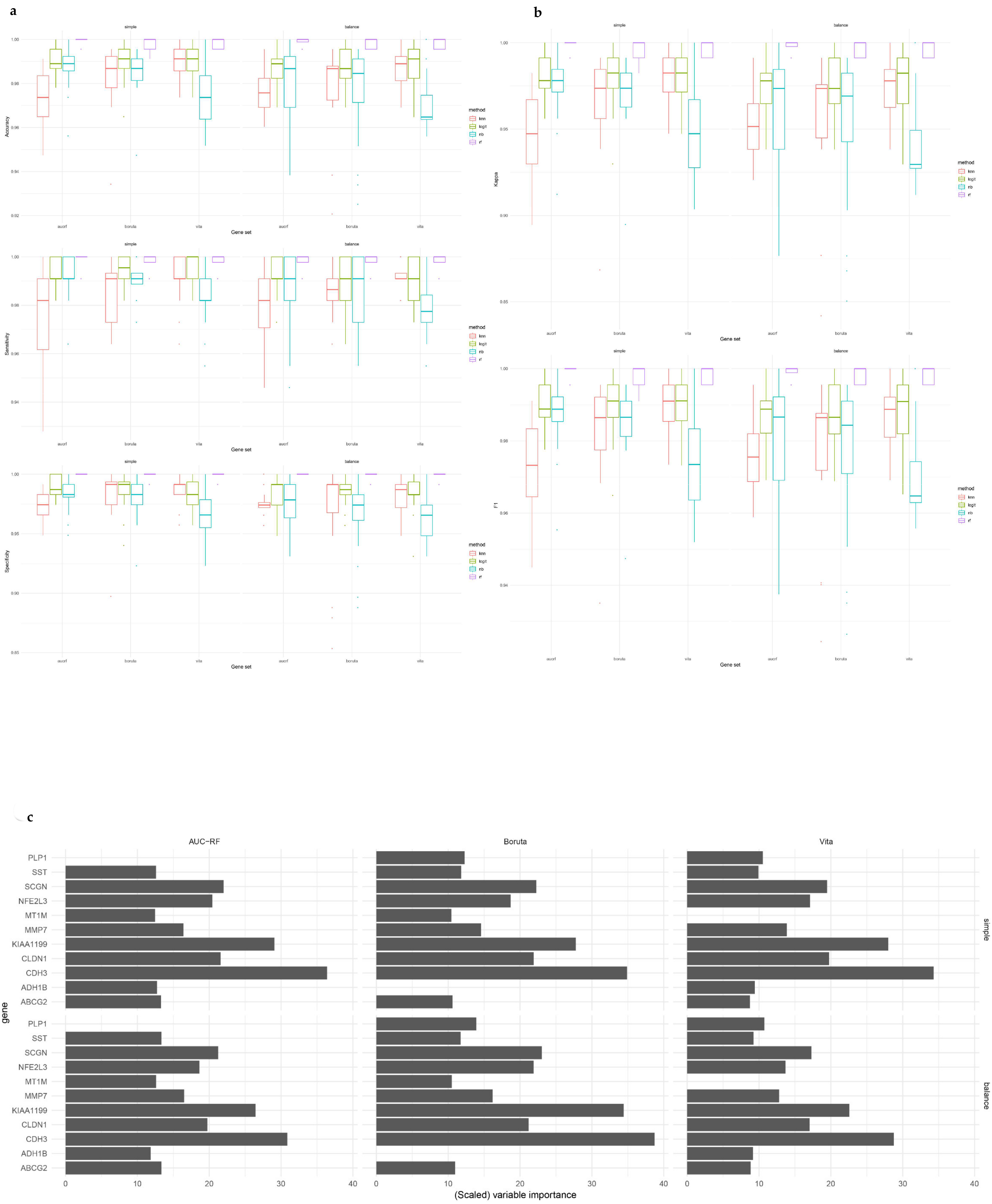

Figure S4: Classification performance of TCGA-derived data sets only and TCGA-derived cancer samples versus GTEx non-cancerous samples.

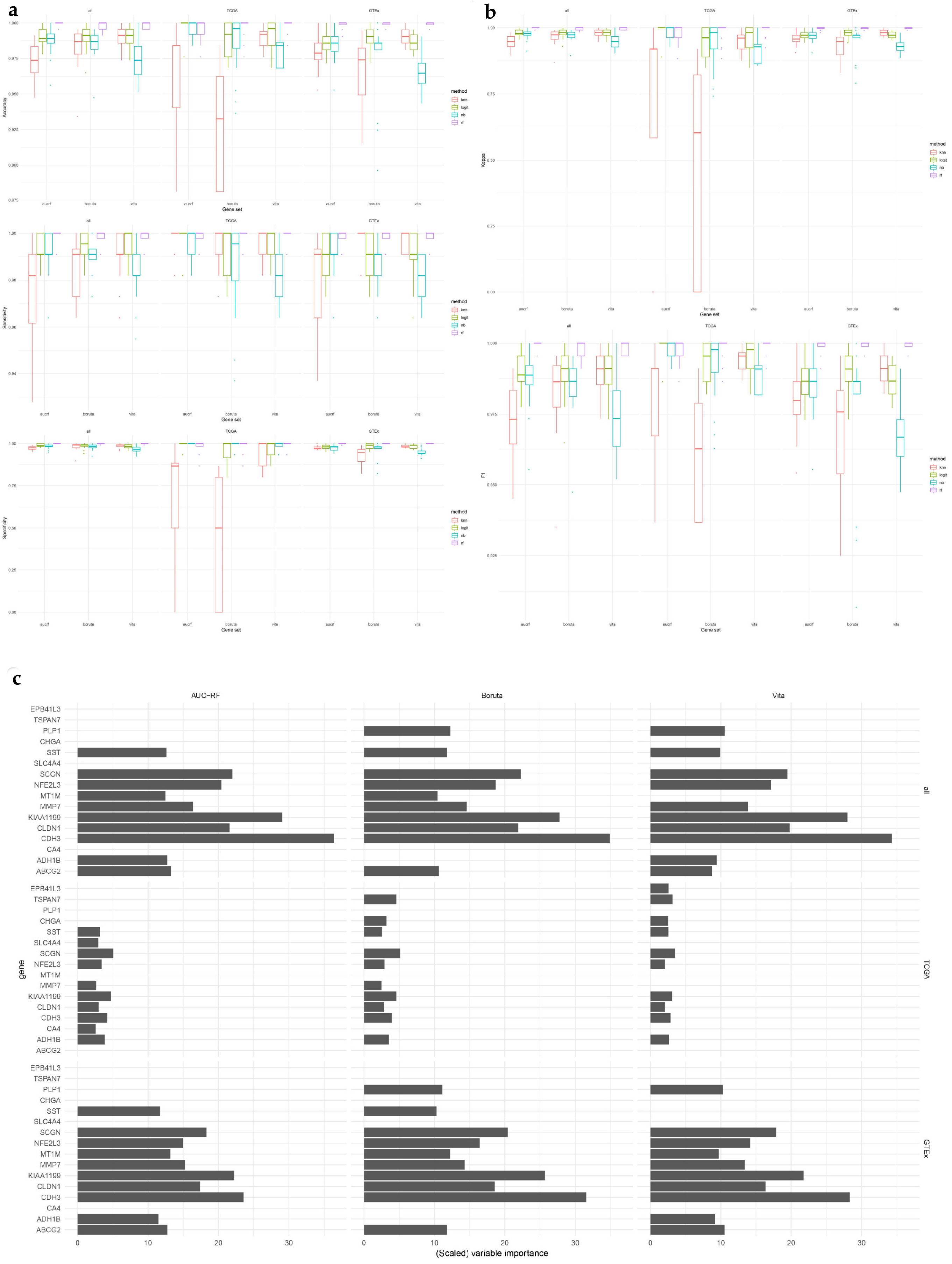

Table S1: Significantly enriched pathways of 19 up-regulated and 53 down-regulated potential biomarkers.

	**Enriched pathways**	**FDR**
**Up-regulated pathways**	Epithelial cell signaling in Helicobacter pylori infection	1.44E-05
	Chemokine signaling pathway	0.0177
	Legionellosis	0.0177
	Cytokine-cytokine receptor interaction	0.0462
	Apoptosis	0.0673
	Pathways in cancer	0.0673
	ECM-receptor interaction	0.0673
	Toll-like receptor signaling pathway	0.0889
**Down-regulated pathways**	Jak-STAT signaling pathway	2.79E-10
	Pathways in cancer	2.84E-09
	Epstein-Barr virus infection	1.10E-08
	Prostate cancer	7.71E-08
	HTLV-I infection	7.71E-08
	Chronic myeloid leukemia	1.54E-07
	Measles	3.58E-05
	Herpes simplex infection	3.58E-05
	Aldosterone-regulated sodium reabsorption	3.58E-05
	Influenza A	4.28E-05
	Acute myeloid leukemia	4.70E-05
	Adipocytokine signaling pathway	8.03E-05
	Osteoclast differentiation	8.03E-05
	Cocaine addiction	9.16E-05
	Neurotrophin signaling pathway	9.16E-05
	Pancreatic cancer	0.000119
	Toxoplasmosis	0.000774
	Amphetamine addiction	0.000774
	Hepatitis C	0.00114
	Colorectal cancer	0.00199
	Leishmaniasis	0.0023
	Cell cycle	0.00379
	Renal cell carcinoma	0.00452
	T cell receptor signaling pathway	0.0061

Insulin signaling pathway	0.0061
Melanogenesis	0.00662
Wnt signaling pathway	0.0076
Transcriptional misregulation in cancer	0.00857
B cell receptor signaling pathway	0.00999
Notch signaling pathway	0.0123
Type II diabetes mellitus	0.0129
Pertussis	0.0168
ErbB signaling pathway	0.017
Chagas disease (American trypanosomiasis)	0.0182
Neuroactive ligand-receptor interaction	0.0194
Bile secretion	0.0226
Bladder cancer	0.0226
Chemokine signaling pathway	0.0256
Glioma	0.031
Long-term potentiation	0.0393
Epithelial cell signaling in Helicobacter pylori infection	0.0408
Salmonella infection	0.041
MAPK signaling pathway	0.041
Tuberculosis	0.0589
Apoptosis	0.063
Amoebiasis	0.0662
Shigellosis	0.0687
NOD-like receptor signaling pathway	0.0752
Vibrio cholerae infection	0.0796
Rheumatoid arthritis	0.0796
Non-small cell lung cancer	0.083
Cytosolic DNA-sensing pathway	0.084
Cholinergic synapse	0.084
Toll-like receptor signaling pathway	0.0883
Vasopressin-regulated water reabsorption	0.0958

Table S2: The summary of Cancer Hallmarks Analytics Tool analysis for 19 up-regulated and 54 down-regulated genes.

Entrez ID	HUGO official name	Invasion and metastasis	Immune destruction	Cellular energetics	Replicative immortality	Evading growth suppressors	Genome instability and mutation	Inducing angiogenesis	Resisting cell death	Sustaining proliferative signaling	Tumor promoting inflammation	Reported in CRC
**4316**	**MMP7**	✓					✓	✓	✓	✓	✓	✓
**57214**	**CEMIP**	✓		✓	✓	✓	✓		✓	✓		✓
**28234**	**SLCO1B3**	✓				✓	✓		✓			✓
**1001**	**CDH3**	✓				✓	✓		✓	✓		✓
**2919**	**CXCL1**	✓	✓			✓		✓	✓	✓	✓	✓
**8140**	**SLC7A5**	✓						✓	✓	✓	✓	✓
**7045**	**TGFBI**	✓			✓	✓	✓	✓	✓	✓	✓	✓
**9603**	**NFE2L3**										✓	✓
**9319**	**TRIP13**	✓				✓	✓				✓	
**9076**	**CLDN1**	✓			✓	✓	✓	✓	✓	✓	✓	✓
**306**	**ANXA3**	✓				✓	✓	✓	✓	✓		
**3576**	**IL8**	✓	✓	✓		✓		✓	✓	✓	✓	✓
**6273**	**S100A2**	✓			✓	✓	✓	✓		✓	✓	
**8549**	**LGR5**	✓		✓	✓	✓	✓	✓	✓	✓		✓
**55165**	**CEP55**					✓	✓			✓		✓
**4319**	**MMP10**	✓	✓			✓	✓	✓	✓	✓	✓	✓
**10874**	**NMU**	✓					✓	✓		✓	✓	
**6373**	**CXCL11**	✓	✓	✓		✓		✓	✓	✓	✓	✓

Query
MMP7: *MMP7 OR Matrix Metallopeptidase 7*
CEMIP: *CEMIP OR KIAA1199 OR Cell Migration Inducing Hyaluronan Binding Protein*
SLCO1B3: *SLCO1B3 OR Solute Carrier Organic Anion Transporter Family Member 1B3*
CDH3: *CDH3 OR Cadherin 3*
CXCL1: *CXCL1 OR C-X-C Motif Chemokine Ligand 1*

SLC7A5: *SLC7A5 OR Solute Carrier Family 7 Member 5*
TGFBI: *TGFBI OR Transforming Growth Factor Beta Induced*
NFE2L3: *NFE2L3 OR Nuclear Factor, Erythroid 2 Like 3*
TRIP13: *TRIP13 OR Thyroid Hormone Receptor Interactor 13*
CLDN1: *CLDN1 OR Claudin 1*
ANXA3: *ANXA3 OR Annexin A3*
IL8: *IL8 OR CXCL8 OR C-X-C Motif Chemokine Ligand 8 OR Monocyte-Derived Neutrophil Chemotactic Factor OR Monocyte-Derived Neutrophil-Activating Peptide OR Granulocyte Chemotactic Protein 1*
S100A2: *S100A2 OR S100 Calcium Binding Protein A2*
LGR5: *LGR5 OR Leucine Rich Repeat Containing G Protein-Coupled Receptor 5*
CEP55: *CEP55 OR Centrosomal Protein 55*
MMP10: *MMP10 OR Matrix Metallopeptidase 10*
NMU: *NMU OR Neuromedin U*
CXCL11: *CXCL11 OR C-X-C Motif Chemokine Ligand 11*

Entrez ID	HUGO official name	Invasion and metastasis	Immune destruction	Cellular energetics	Replicative immortality	Evading growth suppressors	Genome instability and mutation	Inducing angiogenesis	Resisting cell death	Sustaining proliferative signaling	Tumor promoting inflammation	Reported in colon cancer
759	CA1			✓				✓	✓	✓		✓
4499	MT1M	✓						✓	✓			
760	CA2			✓					✓	✓	✓	
57733	GBA3		✓				✓					
1113	CHGA		✓					✓				✓
7166	TPH1						✓	✓		✓	✓	
22802	CLCA4	✓			✓				✓	✓		✓
79686	SYNE3											
3957	LGALS2	✓	✓			✓	✓		✓		✓	
2494	NR5A2			✓		✓	✓		✓	✓	✓	
6387	CXCL12	✓	✓			✓		✓	✓	✓	✓	✓
79799	UGT2A3											
762	CA4			✓		✓	✓	✓	✓	✓		✓
63928	CHP2	✓								✓		
5697	PYY			✓				✓		✓		
1908	EDN3						✓	✓		✓		
5354	PLP1			✓		✓	✓	✓	✓	✓	✓	
343	AQP8	✓		✓				✓	✓	✓	✓	✓
27299	ADAMDEC1		✓						✓	✓		✓
2641	GCG			✓						✓		
10170	DHRS9											
54860	MS4A12									✓		✓
8671	SLC4A4	✓					✓	✓	✓	✓		✓
2981	GUCA2B		✓				✓					✓
126	ADH1C						✓					✓
6338	SCNN1B						✓		✓	✓	✓	

**9073**	**CLDN8**		✓				✓		✓	✓	✓	✓
**54831**	**BEST2**						✓			✓		
**79154**	**DHRS11**											
**270**	**AMPD1**			✓			✓			✓	✓	
**766**	**CA7**						✓			✓		✓
**1114**	**CHGB**							✓		✓		
**608**	**TNFRSF17**									✓	✓	✓
**57126**	**CD177**	✓	✓				✓		✓		✓	
**55532**	**SLC30A10**						✓	✓				✓
**9429**	**ABCG2**	✓				✓	✓	✓	✓	✓		✓
**10590**	**SCGN**					✓			✓	✓		✓
**1836**	**SLC26A2**	✓				✓	✓			✓		
**3294**	**HSD17B2**	✓				✓	✓			✓		✓
**7102**	**TSPAN7**	✓					✓		✓	✓		✓
**125**	**ADH1B**			✓			✓	✓				✓
**6750**	**SST**	✓		✓				✓		✓		✓
**3248**	**HPGD**	✓				✓	✓			✓	✓	✓
**4224**	**MEP1A**							✓			✓	
**1087**	**CEACAM7**		✓						✓		✓	✓
**10022**	**INSL5**									✓		✓
**23136**	**EPB41L3**	✓					✓		✓	✓		
**2908**	**NR3C1**			✓			✓		✓	✓	✓	✓
**11005**	**SPINK5**						✓	✓	✓		✓	
**4692**	**NDN**	✓		✓		✓	✓	✓	✓	✓	✓	✓
**6476**	**SI**					✓		✓		✓		✓
**7367**	**UGT2B17**	✓					✓			✓		
**1811**	**SLC26A3**						✓					✓
**3934**	**LCN2**	✓	✓	✓	✓			✓	✓	✓	✓	

Query
CA1: *CA1 OR Carbonic Anhydrase 1 OR Carbonic Anhydrase I*
MT1M: *MT1M OR Metallothionein 1M*
CA2: *CA2 OR Carbonic Anhydrase 2 OR Carbonic Anhydrase II*
GBA3: *GBA3 OR Glucosylceramidase Beta 3 (Gene/Pseudogene)*
CHGA: *CHGA OR Chromogranin A*
TPH1: *TPH1 OR Tryptophan Hydroxylase 1*
CLCA4: *CLCA4 OR Chloride Channel Accessory 4*
SYNE3: *SYNE3 OR Spectrin Repeat Containing Nuclear Envelope Family Member 3*

LGALS2*: LGALS2 OR Galectin 2*
NR5A2*: NR5A2 OR Nuclear Receptor Subfamily 5 Group A Member 2*
CXCL12*: CXCL12 OR C-X-C Motif Chemokine Ligand 12*
UGT2A3*: UGT2A3 OR UDP Glucuronosyltransferase Family 2 Member A3*
CA4*: CA4 OR Carbonic Anhydrase 4*
CHP2*: CHP2 OR Calcineurin Like EF-Hand Protein 2*
PYY*: PYY OR Peptide YY*
EDN3*: EDN3 OR Endothelin 3*
PLP1*: PLP1 OR Proteolipid Protein 1*
AQP8*: AQP8 OR Aquaporin 8*
ADAMDEC1*: ADAMDEC1 OR ADAM Like Decysin 1*
GCG*: GCG OR Glucagon*
DHRS9*: DHRS9 OR Dehydrogenase/Reductase 9*
MS4A12*: MS4A12 OR Membrane Spanning 4-Domains A12*
SLC4A4*: SLC4A4 OR Solute Carrier Family 4 Member 4*
GUCA2B*: GUCA2B OR Guanylate Cyclase Activator 2B*
ADH1C*: ADH1C OR Alcohol Dehydrogenase 1C (Class I), Gamma Polypeptide*
SCNN1B*: SCNN1B OR Sodium Channel Epithelial 1 Beta Subunit*
CLDN8*: CLDN8 OR Claudin 8*
BEST2*: BEST2 OR Bestrophin 2*
DHRS11*: DHRS11 OR Dehydrogenase/Reductase 11*
AMPD1*: AMPD1 OR Adenosine Monophosphate Deaminase 1*
CA7*: CA7 OR Carbonic Anhydrase 7*
CHGB*: CHGB OR Chromogranin B*
TNFRSF17*: TNFRSF17 OR TNF Receptor Superfamily Member 17*
CD177*: CD177 OR CD177 Molecule OR CD177 Antigen*
SLC30A10*: SLC30A10 OR Solute Carrier Family 30 Member 10*
ABCG2*: ABCG2 OR ATP Binding Cassette Subfamily G Member 2*
SCGN*: SCGN OR Secretagogin, EF-Hand Calcium Binding Protein*
SLC26A2*: SLC26A2 OR Solute Carrier Family 26 Member 2*
HSD17B2*: HSD17B2 OR Hydroxysteroid 17-Beta Dehydrogenase 2*
TSPAN7*: TSPAN7 OR Tetraspanin 7*
ADH1B*: ADH1B OR Alcohol Dehydrogenase 1B (Class I), Beta Polypeptide*
SST*: SST OR Somatostatin*
HPGD*: HPGD OR 15-Hydroxyprostaglandin Dehydrogenase*
MEP1A*: MEP1A OR Meprin A Subunit Alpha*
CEACAM7*: CEACAM7 OR Carcinoembryonic Antigen Related Cell Adhesion Molecule 7*
INSL5*: INSL5 OR Insulin Like 5*
EPB41L3*: EPB41L3 OR Erythrocyte Membrane Protein Band 4.1 Like 3*
NR3C1*: NR3C1 OR Nuclear Receptor Subfamily 3 Group C Member 1*
SPINK5*: SPINK5 OR Serine Peptidase Inhibitor, Kazal Type 5*
NDN*: NDN OR Necdin, MAGE Family Member*
SI*: SI OR Sucrase-Isomaltase*
UGT2B17*: UGT2B17 OR UDP Glucuronosyltransferase Family 2 Member B17*
SLC26A3*: SLC26A3 OR Solute Carrier Family 26 Member 3*
LCN2*: LCN2 OR Lipocalin 2*

Table S3: Survival analysis of 19 up-regulated and 54 down-regulated genes.

	Entrez ID	Offical gene name	OS	DFS
**Up-regulated genes**	3934	*LCN2*	0.86	0.15
	4316	*MMP7*	0.47	0.92
	57214	*KIAA1199 (CEMIP)*	0.78	0.34
	28234	*SLCO1B3*	0.6	0.21
	1001	*CDH3*	0.79	0.37
	2919	*CXCL1*	0.06	0.49
	8140	*SLC7A5*	0.9	0.62
	7045	*TGFBI*	0.69	0.016
	9603	*NFE2L3*	0.41	0.55
	9319	*TRIP13*	0.14	0.081
	9076	*CLDN1*	0.66	0.17
	306	*ANXA3*	0.009	0.15
	3576	*IL8 (CXCL8)*	0.032	0.28
	6273	*S100A2*	0.45	0.044
	8549	*LGR5*	0.2	0.095
	55165	*CEP55*	0.32	0.47
	4319	*MMP10*	0.087	0.44
	10874	*NMU*	0.27	0.23
	6373	*CXCL11*	0.61	0.0096
**Down-regulated genes**	2690	*GHR*	0.89	0.22
	759	*CA1*	0.45	0.38
	4499	*MT1M*	0.68	0.46
	760	*CA2*	0.076	0.16
	57733	*GBA3*	0.12	0.13
	1113	*CHGA*	0.14	0.29
	7166	*TPH1*	0.73	0.87
	22802	*CLCA4*	0.056	0.65
	79686	*SYNE3*	0.27	0.33
	3957	*LGALS2*	0.21	0.97
	2494	*NR5A2*	0.029	0.38
	6387	*CXCL12*	0.4	0.086
	79799	*UGT2A3*	0.13	0.86
	762	*CA4*	0.055	0.25
	63928	*CHP2*	0.18	0.66
	5697	*PYY*	0.15	0.15
	1908	*EDN3*	0.16	0.24
	5354	*PLP1*	0.66	0.064
	343	*AQP8*	0.019	0.46
	27299	*ADAMDEC1*	0.025	0.54
	2641	*GCG*	0.0063	0.37
	10170	*DHRS9*	0.18	0.54

54860	*MS4A12*	0.19	0.21
8671	*SLC4A4*	0.033	0.76
2981	*GUCA2B*	0.33	0.63
126	*ADH1C*	0.2	0.6
6338	*SCNN1B*	0.23	0.11
9073	*CLDN8*	0.75	0.46
54831	*BEST2*	0.00094	0.16
79154	*DHRS11*	0.24	0.29
270	*AMPD1*	0.15	0.28
766	*CA7*	0.53	0.61
1114	*CHGB*	0.33	0.22
608	*TNFRSF17*	0.25	0.084
57126	*CD177*	0.038	0.21
55532	*SLC30A10*	0.48	0.91
9429	*ABCG2*	0.81	0.79
10590	*SCGN*	0.56	0.64
1836	*SLC26A2*	0.099	0.84
3294	*HSD17B2*	0.8	0.36
7102	*TSPAN7*	0.66	0.55
125	*ADH1B*	0.8	0.77
6750	*SST*	0.91	0.11
3248	*HPGD*	0.65	0.62
4224	*MEP1A*	0.68	0.57
1087	*CEACAM7*	0.029	0.12
10022	*INSL5*	0.7	0.74
23136	*EPB41L3*	0.45	0.12
2908	*NR3C1*	0.61	0.068
11005	*SPINK5*	0.86	0.17
4692	*NDN*	0.56	0.16
6476	*SI*	0.56	0.64
7367	*UGT2B17*	0.13	0.82
1811	*SLC26A3*	0.003	0.58
